# Method for developing a sub-10 fs ultrafast electron diffraction technology

**DOI:** 10.1063/4.0000012

**Published:** 2020-06-09

**Authors:** Hyun Woo Kim, In Hyung Baek, Junho Shin, Sunjeong Park, Hyeon Sang Bark, Key Young Oang, Kyu-Ha Jang, Kitae Lee, Nikolay Vinokurov, Young Uk Jeong

**Affiliations:** 1 Radiation Center for Ultrafast Science, Korea Atomic Energy Research Institute, 989-111 Daedeok-Daero, Yuseong-gu, Daejeon, South Korea; 2 Budker Institute of Nuclear Physics, Lavrent'yeva, 11, 630090 Novosibirsk, Russia

## Abstract

The experimental observation of femtosecond dynamics in atoms and molecules by stroboscopic technologies utilizing x ray or electron flashes has attracted much attention and has rapidly developed. We propose a feasible ultrafast electron diffraction (UED) technology with high brightness and a sub-10 fs temporal resolution. We previously demonstrated a UED system with an overall temporal resolution of 31 fs by using an RF photoelectron gun and a 90° achromatic bending structure. This UED structure enabled a bunch duration of 25 fs and a low timing jitter of less than 10 fs while maintaining a high bunch charge of 0.6 pC. In this paper, we demonstrate a simple way to further compress the electron bunch duration to sub-10 fs based on installing an energy filter in the dispersion section of the achromatic bend. The energy filter removes the electrons belonging to nonlinear parts of the phase space. Through numerical simulations, we demonstrate that the electron bunches can be compressed, at the sample position, to a 6.2 fs (rms) duration for a 100 fC charge. This result suggests that the energy filtering approach is more viable and effective than complicated beam-shaping techniques that commonly handle the nonlinear distribution of the electron beam. Furthermore, a gas-filled hollow core fiber compressor and a Ti:sapphire amplifier are used to implement pump laser pulses of less than 5 fs (rms). Thus, we could present the full simulation results of a sub-10 fs UED, and we believe that it will be one of the technical prototypes to challenge the sub-fs time resolution.

## I. INTRODUCTION

Understanding the ultrafast atomic and molecular dynamics in matter has generated a great amount of interest in recent years. The development of x-ray free-electron lasers (XFELs)^1–5^ and ultrafast electron diffraction (UED)^6–16^ enabled the direct observation of atomic and molecular structure dynamics with sub-nanometer and femtosecond-scale resolutions. XFELs have revealed a new horizon for ultrafast x-ray science by generating intense femtosecond pulses in the soft and hard x-ray regions of the spectrum.^1–5^ In particular, the femtosecond time resolution provided by XFELs has opened the opportunity to study the role and importance of ultrafast dynamics in physics, chemistry, and biology, which have been challenging to approach before.^17–25^ Several breakthrough results in this field have been produced since the first XFEL, the Linac Coherent Light Source (LCLS),^1,17^ was put into operation 10 years ago. UED uses electron pulses with much lower energy and charge than the XFEL pulses, being suitable for usage in a larger number of laboratories^6–16^ and complementing the XFEL in the study of femtosecond time-resolved structural dynamics of thin samples and small molecules.

Recently, the UED technology has been competing with XFEL for providing a shorter time resolution.^15,16^ The XFEL pulse duration has already been reduced to the attosecond range,^26^ but its timing jitter reduction is difficult due to the large physical size of the facilities hosting such equipment.^22^ In particular, implementing a timing jitter of less than 10 fs (rms) is very challenging. In contrast, the UED timing jitter has been successfully reduced to less than 10 fs (rms) using an RF photoelectron gun and a 90° bend structure.^15^ Encouragingly, this technique seems to facilitate a further reduction of the timing jitter to the attosecond scale.^15^ Obtaining electron bunches that are at once ultra-short, time-stable, and high-charge is difficult due to space-charge force. However, low-energy sub-10-fs electron bunches have been successfully generated by ballistic bunching with RF buncher cavities^27–29^ and bunch compression using the electric field of THz^30,31^ or IR pulses.^32^ As an alternative concept, a laser-plasma-based electron source for sub-10 fs electron diffraction was proposed.^33^ Techniques for sub-fs electron beam generation by correcting the 2^nd^ order distortion with higher harmonic RF cavities^34^ and techniques for improving the laser-microwave synchronization by a microwave phase feedback system^35^ have also been evaluated. However, it remains challenging to develop technologies to achieve high scattering power, sub-10 fs timing stability, and sub-10 fs temporal pulse width simultaneously.

In this paper, we propose an UED device with an overall temporal resolution of less than 10 fs (rms) using an electron beam with a charge of 100 fC or more, providing an effective beam brightness similar to that of the XFEL. To achieve such unprecedented temporal resolution, the optical pump pulse width, timing stability, and electron bunch duration need to be optimized. It is expected that a pump laser pulse duration of less than 5 fs (rms) and an energy of ∼1 mJ, respectively, can be obtained by adding a gas-filled-hollow-fiber compressor to a Ti:sapphire laser amplifier. Regarding the timing jitter, we established the jitter-free condition and demonstrated the timing jitter of less than 10 fs through the direct measurement.^15^ In this paper, we demonstrate a simple method to obtain ultrashort electron bunches of less than 10 fs (rms) by using an energy filter in the dispersion region of the 90° bend of the Radiation Center for Ultrafast Science (RCUS) UED,^15^ at the Korea Atomic Energy Research Institute (KAERI). Furthermore, we performed beam dynamics simulations using ASTRA.^36^ The numerical results confirmed that the electron beam can be compressed up to 6.2 fs (rms) for a bunch charge of 100 fC, even for initial beams with Gaussian distributions in time and space. This method is more effective in controlling the space-charge effect than the complicated methods of beam shaping in space and time. Note that the space-charge effect limits the degree of bunch compression that can be reached. Taking into consideration all the factors mentioned, a sub-10-fs UED can be obtained using an RF photogun and a 90° achromatic bending structure, which might be a promising method to challenge the UED with an attosecond temporal resolution.

## II. METHODS AND RESULTS

### A. Overall strategy for sub-10 fs UED technology

While implementing the sub-10 fs UED, we considered two practical points for determining the minimum bunch charge. First, the temporal characteristics of each electron bunch should be measurable. The bunch duration and time-of-arrival deviation are the two most critical temporal characteristics to be analyzed. However, the shot-to-shot identification of the temporal characteristic is challenging due to the very low energy and low charge of the electron bunch. The improvement of the bunch duration and temporal stability is possible only if single-pulse measurements are available. The second practical point, challenging for user experiments, is to maintain a scattering measurement performance similar to that of the XFEL, which, currently, is the most advanced technology available for ultrafast dynamics. The most critical issue is keeping the bunch charge larger than 100 fC. The scattering power of the electrons is 10^5^–10^6^ times stronger than that of the x rays, which are strongly dependent on the electron energy. Since XFELs have 10^11^–10^12^ photons per pulse, the minimum bunch charge might be of the order of 100–200 fC, corresponding to approximately 10^6^ electrons per pulse. This value is consistent with the current performance of single-shot THz streaking measurements. Consequently, we impose the constraint that the sub-10 fs UED bunch charge exceeds 100 fC.

The overall UED temporal resolution, Δτ, is given by the following equation:

$$\Delta \tau ≅\sqrt{\Delta \tau_{\mathrm{laser}}^{2}+\Delta \tau_{e-\mathrm{beam}}^{2}+\Delta \tau_{\mathrm{jitter}}^{2}+\Delta \tau_{\mathrm{drift}}^{2}},$$ (1)

where the velocity mismatch between the pump laser and electron pulses was neglected. $\Delta \tau_{\mathrm{laser}}$ is the laser pulse temporal duration, $\Delta \tau_{e-\mathrm{beam}}$ is the electron pulse temporal duration, $\Delta \tau_{\mathrm{jitter}}$ is the arrival timing jitter between the pump laser and probe electron pulses at the sample, and $\Delta \tau_{\mathrm{drift}}$ is the slow time drift between the laser and electron pulses. When the electron energy is a few MeV and the laser and electron pulses are collinearly incident on a thin sample, the velocity mismatch between the laser and electron beams is small enough to be ignored.

**FIG. 1.**
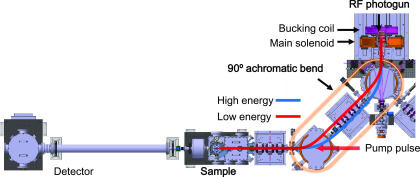
Layout of the RCUS UED facility. The red and blue lines delineate trajectories of electrons with different energies.

For achieving an overall temporal resolution of less than 10 fs (rms), each of the four terms involved in the above equation have to be under 10 fs (rms). In a previous paper, we demonstrated that the timing jitter, although limited by the accuracy of the measurement, can be reduced to 7.8 fs (rms).^15^ We confirmed, through a crossing experiment, that a less than 2 fs (rms) of the timing jitter can be obtained. Our ASTRA simulation results confirmed that, with the RCUS UED, achieving a timing jitter below 1 fs (rms) is certainly possible. Furthermore, we studied the impact of the timing drift of our device on the temporal resolution. Assuming that the achromatic beam path at the 90° bending structure is time-invariant over a long period of time, the timing drift is compensated by the same mechanism as the timing jitter. Let us consider a positive sign of the timing error when the electron beam arrives at the sample earlier than the laser pulse. The timing error could be then expressed as

$$\Delta \tau_{\mathrm{error}}\equiv T_{\mathrm{laser}}^{\mathrm{sample}}-T_{e-\mathrm{beam}}^{\mathrm{sample}},$$ (2)

where $T_{\mathrm{laser}}^{\mathrm{sample}}$ and $T_{e-\mathrm{beam}}^{\mathrm{sample}}$ are the arrival times of the pump laser and electron pulses at the sample. The timing jitter represents the fast timing deviation, and the timing drift represents the slow timing changeover a long period of time. The timing delay of the electron beam at the sample can be expanded as

$$T_{e-\mathrm{beam}}^{\mathrm{sample}}=\frac{l_{e}}{v_{e}}+\alpha \left(T_{RF}^{\mathrm{gun}}-T_{\mathrm{laser}}^{\mathrm{gun}}\right)+\frac{l_{\mathrm{gun}}}{c},$$ (3)

where $l_{e}$ is the flight distance of the electron beam after the gun, $v_{e}$ is the velocity of the electron beam, $T_{\mathrm{laser}}^{\mathrm{gun}}$ and $T_{RF}^{\mathrm{gun}}$ are the arrival times of the UV laser and the target RF phase at the gun surface, α is the jitter compensation ratio, $l_{\mathrm{gun}}$ is the propagation distance of the laser pulses from the beam splitter to the gun surface, and c is the speed of light. The beam splitter divides the laser pulse into two pulses, which propagate to the gun surface and the sample; therefore, timing delay before the beam splitter is a common mode and, thus, neglected in $l_{\mathrm{gun}}$. The term $l_{e}v_{e}^{-1}$ represents the travel time of the electron beam, $\alpha \left(T_{RF}^{\mathrm{gun}}-T_{\mathrm{laser}}^{\mathrm{gun}}\right)$ describes the timing change from the gun after the jitter compensation mechanism, and $l_{\mathrm{gun}}c^{-1}$ shows the optical timing delay from the beam splitter to the gun. Notably, the second term involves the timing jitter of the active synchronization^37^ between the laser and the RF oscillator, while the other terms are dominated by the slow drift. The timing of the laser pulse at the sample location is

$$T_{\mathrm{laser}}^{\mathrm{sample}}=\frac{l_{\mathrm{pump}}}{c},$$ (4)

where $l_{\mathrm{pump}}$ is the laser pulse flight distance from the beam splitter to the sample. Summing all terms together, the timing drift of the electron beam at the sample position becomes

$$\Delta \tau_{\mathrm{error}}=\alpha \Delta \tau_{\mathrm{gun}}+\Delta \tau \prime ,$$ (5)

where $\Delta \tau_{\mathrm{gun}}=T_{\mathrm{laser}}^{\mathrm{gun}}-T_{RF}^{\mathrm{gun}}$ and $\Delta \tau^{\prime }=\frac{l_{\mathrm{pump}}}{c}-(\frac{l_{e}}{v_{e}}+\frac{l_{\mathrm{gun}}}{c})$. The term $\alpha \Delta \tau_{\mathrm{gun}}$ represents the jitter suppression mechanism effect on the drift. Based on numerical simulations using the 90° bending structure shown in Fig. 1, we expect for α a value of 0.1. Such a low α value implies that the timing drift of the electron beam at the sample is much smaller compared to the timing drift of the gun. The second term of Eq. (5), Δτ′, represents the timing difference between the two beam paths after the beam splitter. The timing difference can be reduced to a 10 fs scale by collocating the beam paths on the same optical table, carefully matching the path lengths.^38^ The total drift for a 1 h time interval, approximately 300 fs, was caused by a change in temperature of approximately 1 °C between the laboratory and klystron rooms. With a repetition rate of 50 Hz, a typical user application requires less than 60 s of acquisition time, and the timing drift is proportional to the acquisition time. Since there are ongoing attempts to reduce both the acquisition time by increasing the pulse repetition rate and temperature fluctuation of the facility, the timing drift during a single set of data acquisition could soon reach the scale of a few fs.

For observing the rapid recovery dynamics of atomic or molecular structures after impulsive excitation, it is natural that a pulse duration of the pumping laser should be as short as possible. Considering technical limits and a user convenience, few-cycle near-infrared pulses with millijoule-class energy and a stable (<60 mrad) carrier envelope phase (CEP) can be suitable to bring out the best performance of sub-10 fs UED technology as an optical pumping source. A CEP-stabilized Ti:sapphire laser amplifier combined with a hollow-core fiber (HCF) compressor^39,40^ satisfies all prerequisites mentioned above. Nowadays, it is commercially available.

Therefore, the main task required to reach sub-10 fs temporal resolution using the 90° bending UED is to generate electron bunches shorter than 10 fs (rms). In Sec. II B, we evaluate possible ways of obtaining sub-10 fs electron bunches, with charges of 100 fC or larger, at the sample position.

### B. Sub-10 fs electron beam compression using an energy filter

The RCUS UED beamline consists of an S-band RF photoelectron gun^41^ and two 90° achromatic bends (Fig. 1), which provide the bunch compression and timing jitter suppression simultaneously, as follows. A several tens to a few hundred femtoseconds long ultraviolet (UV) laser pulse illuminates a copper photocathode. The cathode emits electrons, which are subsequently accelerated by the electric field in the RF gun, repelling each other due to space-charge forces. Thus, the electron bunch arrives at the bend with the high-energy electrons traveling at the front and the low-energy electrons at the end. However, their trajectories inside the bend depend on their energy, and the high-energy electrons exchange locations with the low-energy electrons by the time they exit the bend. Then, the high-energy electrons catch up with the low-energy electrons while traveling through a straight beamline toward the sample. Thus, the compressed electron bunch duration was measured to be 25 fs (rms) at the sample, for a bunch charge of 0.6 pC.^15^

For achieving a ballistic compression of the electron bunch at the sample by using the 90° achromatic bend, electron bunches with a negative energy chirp of approximately –60 eV/fs at the entrance of the bend are required.^15^ We obtained a negative energy chirp in the longitudinal space-charge field by using an electron bunch with the shape of a thin pancake. The optimized longitudinal space-charge field at the exit of the RF gun was approximately 10 kV/m. The field strength of the space-charge field is determined by the volume and charge of the electron bunch, which can be controlled by varying the intensity, spot size, and pulse duration of the UV laser pulse and the strength of the main focusing solenoid of the gun. To investigate the conditions the electron beam has to satisfy for achieving sub-10 fs electron bunches at the sample, we set the diameter and minimum charge of the bunch at the sample to 1.5 mm and 100 fC, respectively. The electron beam is focused on a scintillator screen located 2 m away from the sample, to obtain the best images of the UED patterns.

**FIG. 2.**
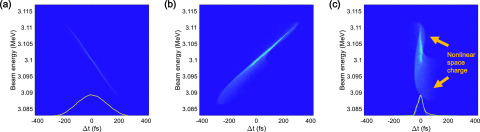
Simulated longitudinal phase space of a Gaussian electron bunch with a bunch charge of 1 pC at the (a) entrance of the 90° achromatic bend, (b) end of the 90° achromatic bend, and (c) sample. Orange lines in the inset [(a) and (c)] indicate the longitudinal profile of the electron bunch, and the cyan line (c) indicates a Gaussian fitting curve. The bunch duration at the sample is 27.2 fs (rms).

When the intensity of the laser pulse is much lower than the value required for photo-emission saturation, the photoelectron beam distribution follows that of the laser pulse, which is close to a Gaussian distribution in space and time. The Gaussian electron beam has a nonlinear component in the space-charge field, which increases the emittance and duration of the bunch. The normalized transverse emittance of the electron beam with a bunch charge of 1 pC and a beam energy of 3.1 MeV increased, at the sample location, from the thermal emittance value of 0.14 mm mrad to 0.32 mm mrad. The longitudinal phase space of the Gaussian electron bunch at the locations of the gun exit, end of the bend, and sample is shown in Fig. 2. One can clearly see the distortion of the phase space distribution of the electron bunch traveling through the bend and the straight sections. The electrons traveling in the longitudinal head and tail parts of the distribution are produced by the nonlinear space-charge field, contributing to the increase in bunch duration. Therefore, to further compress the electron bunch after inducing the negative energy chirp (i.e., passing through the 90° achromatic bend), the space-charge effect on the electron bunch has to be minimized. An obvious and simple solution for suppressing the space-charge induced degradation of the bunch compression is the reduction of the bunch charge.

**FIG. 3.**
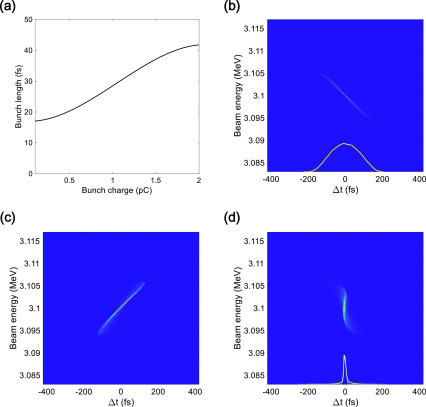
(a) Simulated minimum electron bunch duration as a function of bunch charge for a Gaussian distribution. Simulated longitudinal phase space of a Gaussian electron bunch with a bunch charge of 100 fC at (a) the entrance of the 90° achromatic bend, (b) the end of the 90° achromatic bend, and (c) the sample. Orange lines in the inset [(b) and (d)] indicate the longitudinal profile of the electron bunch, and the cyan line (d) indicates a Gaussian fitting curve. The bunch duration at the sample is 17.1 fs (rms).

We simulated the bunch duration of the electron beam along the beamline, from the gun to the sample location, where the maximum temporal compression of the bunch is achieved. The initial bunch duration was 55 fs (rms). When the initial bunch duration was longer than 55 fs (rms), the compressed bunch duration increased due to the effect of the RF curvature on the charges. In the ASTRA simulations, we kept the initial bunch duration, 55 fs (rms), unchanged and scanned the UV spot size from 0.072 to 0.212 mm (rms) while varying the main solenoid strength from 0.15 to 0.195 T and the bunch charge from 0.1 to 2.0 pC. The compressed bunch duration dependence on the bunch charge at the sample is presented in Fig. 3(a), and the longitudinal phase space of the Gaussian electron bunch with 100 fC charge at the entrance of the bend, bend end, and sample is presented in Figs. 3(b)–3(d). According to our results, an electron bunch with a bunch charge of 100 fC could be compressed to 17.1 fs (rms). However, we could not achieve a sub-10-fs bunch due to the electrons produced by the nonlinear space-charge field in the longitudinal head and tail parts of the distribution, as shown in Fig. 3(d).

**FIG. 4.**
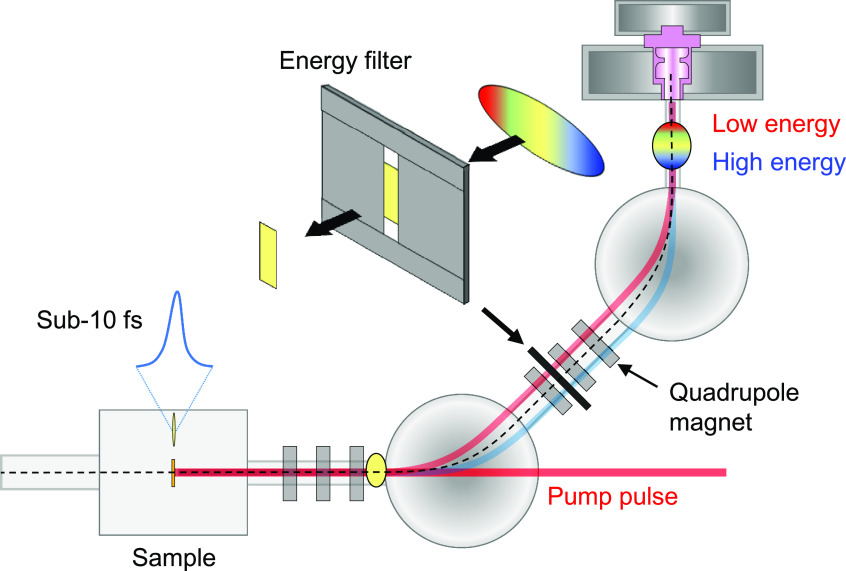
Generation of the sub-10 fs electron bunch via the cutoff electron beams using an energy filter in the dispersion section.

After passing by the first bending magnet, the electrons align on the horizontal axis according to their energy due to horizontal dispersion. Therefore, the longitudinal head and tail parts can be removed by installing an energy filter in the dispersion section of the achromatic bend. The final energy and energy spread of the electron bunch can be selected by adjusting the position and width of the energy filter in the dispersion section, respectively. We determined the position of the energy filter in a section near the highest dispersion, between the second and third quadrupoles after the first bending magnet, as shown in Fig. 4.

The horizontal dispersion at the energy filter, $\eta_{x}$, is given by

$$\eta_{x}=\rho \left(1-\text{cos}\;\theta \right)+L\text{sin}\left(\theta \right)\approx 0.43\;m,$$ (6)

where ρ is the curvature radius of the first bending magnet, θ is a bending angle, and *L* is the drift length from the exit of the first bending magnet to the energy filter. The horizontal displacement for momentum deviation Δp/p is given by

$$\Delta x(z)=\eta_{x}(z)\Delta p/p.$$ (7)

**FIG. 5.**
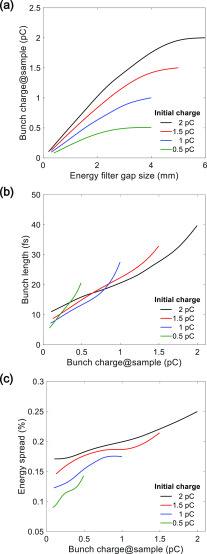
(a) Simulated bunch charge as a function of width of the energy filter. (b) Simulated bunch duration as a function of bunch charge. (c) Simulated energy spread as a function of bunch charge. The bunch charge is varied horizontally [(b) and (c)] by adjusting the width of the energy filter.

**FIG. 6.**
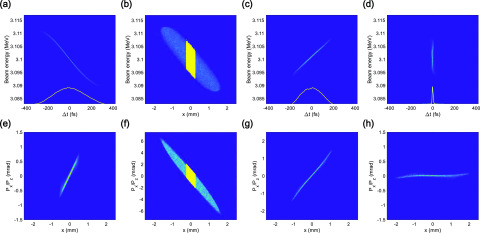
Simulated longitudinal [(a), (c), and (d)] and transverse [(e)–(h)] phase space for a Gaussian distribution electron bunch with a bunch charge of 500 fC at the [(a) and (e)] entrance and [(c) and (g)] end of the 90° achromatic bend and at [(d) and (h)] the sample. Simulated phase space (x–E plane) (b) and transverse phase space (f) at the energy filter. The yellow dots indicate the electrons that pass through the energy filter. Orange lines in the inset [(a), (c), and (d)] indicate the longitudinal profile of the electron bunch, and the cyan line (d) indicates a Gaussian fitting curve. The bunch charge after the energy filter is 0.1 pC. The bunch duration at the sample is 6.2 fs (rms).

Only electrons with horizontal displacements matching the energy gap of the filter can pass through. The bunch charge dependence on the width of the filter is presented in Fig. 5(a). The energy spread and the bunch duration at the sample depend on the width of the filter, as shown in Figs. 5(b) and 5(c). The minimum bunch duration can be achieved when the initial bunch charge and the width of the energy filter are 500 fC and 500 *μ*m, respectively. The energy spread and the bunch charge declined by 38% and 80%, respectively, after passing through the filter. In Fig. 6, the longitudinal and transverse phase space of the electron bunch after passing through an energy filter with a width of 500 *μ*m and an initial charge of 500 fC is presented. The energy chirp at the entrance of the 90° achromatic bend displays the longitudinal head and tail parts of the electron distribution [Fig. 6(a)], which are subsequently removed by the energy filter, as shown in Fig. 6(b). After passing through the 90° achromatic bend, the energy chirp is almost linear, as shown in Fig. 6(c), and the electron bunch can be further compressed to 6.2 fs (rms) at the sample position, as shown in Fig. 6(d). The dispersion is completely compensated, and the electron bunch is collimated at the sample, as shown in Fig. 6(h). The normalized horizontal emittance without and with an energy filter are 0.198 mm mrad and 0.189 mm mrad, respectively.

## III. DISCUSSION

A uniformly filled ellipsoidal (UE) distribution has been proposed as the ideal distribution that can prevent the deterioration in beam quality due to the nonlinear space-charge field.^42,43^ The emittance at the sample can be kept close to the thermal emittance. Furthermore, the formation of the longitudinal head and tail parts due to the nonlinear space-charge can be prevented. However, because the electrons propagate through a 3.1 m long beamline from the photocathode to the sample, it is difficult to preserve the UE distribution.

**FIG. 7.**
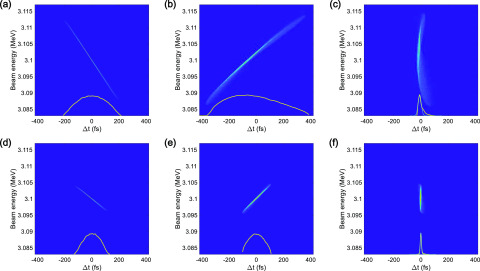
Simulated longitudinal phase space of the UE distribution electron bunch with bunch charges of 1 pC and 200 fC at the entrance [(a) and (d)] and the end [(b) and (e)] of the 90° achromatic bend and at the sample [(c) and (f)]. The inset lines indicate the longitudinal profile of the electron bunch (orange) and the Gaussian fitting curve (cyan) [(c) and (f)]. The bunch duration of the electron bunch with the bunch charges of 1 pC and 100 fC are 18.9 fs (rms) and 6.9 fs (rms), respectively.

**FIG. 8.**
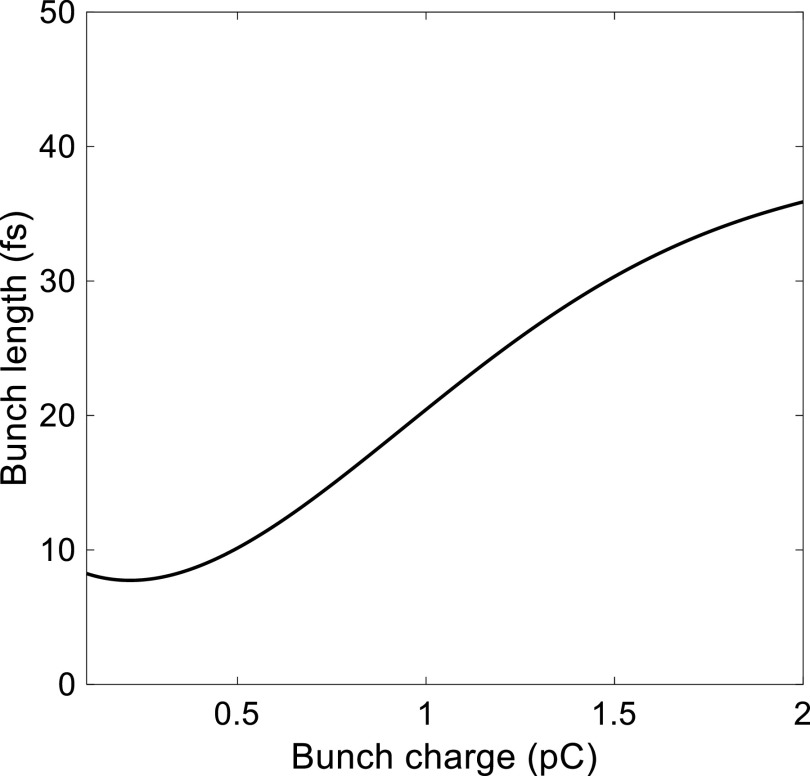
Simulated minimum electron bunch duration as a function of bunch charge for a UE distribution.

Figures 7(a) and 7(d) show the longitudinal phase space of the UE electron bunch at the entrance of the 90° achromatic bend, for bunch charges of 1 pC and 200 fC, respectively. Even if the initial electron bunch has a UE distribution, its longitudinal distribution may change into a Gaussian shape as it passes through the 90° achromatic bend. After exiting the bend, the electron bunch propagates through a final 1.2 m long, straight beamline before reaching the sample. At this stage, the electron bunch is focused in the longitudinal direction and thus affected by the strong space-charge field, which produces longitudinal head and tail parts in the bunch distribution, as shown in Figs. 7(b) and 7(c). The emittance of a 1 pC electron bunch increases from 0.14 mm mrad to 0.18 mm mrad, with a bunch duration of 18.9 fs (rms) at the sample. From the shape of the longitudinal phase space of the 200 fC UE electron bunch presented in Figs. 7(e) and 7(f), it is clear that the longitudinal distribution is preserved after passing through the 90° achromatic bend. The electron bunch is further compressed up to 6.9 fs (rms) at the sample, which is the minimum bunch duration achieved by the UE electron beam while changing the bunch charge from 0.1 to 2 pC, as shown in Fig. 8.

The energy fluctuations of the electron beam create the horizontal position fluctuations at the energy filter location. Energy fluctuations are the main cause of timing jitter between the pump pulse and the electron beam and can cause charge fluctuations. As mentioned in Sec. II, to reduce the temporal resolution to less than 10 fs (rms), the timing jitter and drift, as well as the bunch duration, should not exceed 5 fs (rms). The timing jitter is caused by beam arrival time fluctuations due to changes in the electron beam energy induced by the fluctuations of the laser injection phase and the RF amplitude. To compensate the timing jitter to sub-fs duration, the electron beam energy and the laser injection phase have to be 3.1 MeV and 1.9° from the on-crest where the electron beam is at maximum energy. The measured jitter of the laser injection phase to the RF of the gun was approximately 40 fs (rms).^15^ The energy fluctuation corresponding to a laser injection phase jitter of 40 fs is 59 eV. Finally, we reduced the arrival timing jitter at the sample to 0.3 fs (rms).

**FIG. 9.**
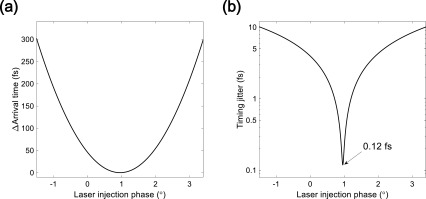
(a) Simulated arrival time difference at the energy filter as a function of the laser injection phase. (b) Simulated timing jitter at the sample for a 40 fs (rms) laser injection phase jitter.

The electron beams passing through the energy filter have the same energy. Thus, the 90° achromatic bend cannot perfectly compensate for the timing jitter under the previous conditions. The laser injection phase needs to be optimized so that the arrival time of the electron beam at the energy filter does not depend on the laser injection phase jitter. The arrival time difference at the energy filter as a function of the laser injection phase is presented in Fig. 9(a), showing that the arrival time fluctuation reaches a minimum for a laser injection phase of 0.976°. At the energy filter, the timing jitter corresponding to the 40 fs (rms) laser injection phase jitter is as low as 0.12 fs (rms) for a laser injection phase of 0.976°. After exiting the 90° achromatic bend, the electron beams travel equal trajectories to the sample with the same velocity. The timing jitter at the energy filter and the sample locations is the same, as shown in Fig. 9(b). The energy fluctuation due to the 40 fs (rms) laser injection phase jitter is 44 eV and causes a horizontal position fluctuation of 0.185 nm at the energy filter. The electron beam diameter at the energy filter is approximately 3.2 mm, and the width of the energy filter is 500 *μ*m. Assuming that the horizontal position fluctuation at the energy filter is ∼10 *μ*m based on our measurements, it causes the arrival timing jitter of ∼0.2 fs and the bunch duration difference of ∼30 fs.

## IV. CONCLUSION

The key technologies with application in time-resolved ultrafast experiments using electron beams require the ability to generate short electron beams and a timing synchronization between the electron beams and the pump laser pulses. The RCUS UED employs 90° achromatic bends as magnetic electron bunch compressors. The dispersion produced by a bending magnet arranges the electrons along the horizontal direction in an order determined by their energy. Here, we describe a simple and effective method for generating sub-10 fs electron bunches based on an energy filter placed at a dispersion section in the achromatic bend. The electrons affected by the nonlinear space-charge field can be eliminated by using the slit-shaped energy filter. The electron beam energy and energy spread can be adjusted by varying the position and gap size of the energy filter, respectively. The truncated electron beams can be compressed up to 6.2 fs (rms) at the sample. The proposed bunch compression scheme is a promising method to generate sub-10-fs electron beams with a timing jitter in the attosecond range, which can open the way for UED technology with sub-10 fs overall temporal resolution.

## Data Availability

The data that support the findings of this study are available from the corresponding author upon reasonable request.
